# Prognostic Factors Involved in the Epithelial–Mesenchymal Transition Process in Colorectal Cancer Have a Preponderant Role in Oxidative Stress: A Systematic Review and Meta-Analysis

**DOI:** 10.3390/cancers12113330

**Published:** 2020-11-11

**Authors:** Eva Parisi, Anabel Sorolla, Robert Montal, Rita González-Resina, Anna Novell, Antonieta Salud, Maria Alba Sorolla

**Affiliations:** 1 Research Group of Cancer Biomarkers, Biomedical Research Institute (IRBLleida), 25198 Lleida, Spain; eparisi@irblleida.cat (E.P.); rmontal.lleida.ics@gencat.cat (R.M.); rgr5@alumnes.udl.cat (R.G.-R.); anovell@xtec.cat (A.N.); masalud.lleida.ics@gencat.cat (A.S.); 2 Harry Perkins Institute of Medical Research, QEII Medical Centre, Nedlands, WA 6009, Australia; anabel.sorollabardaji@perkins.uwa.edu.au; 3 Centre for Medical Research, The University of Western Australia, Crawley, WA 6009, Australia; 4 Department of Medical Oncology, Arnau de Vilanova University Hospital, 25198 Lleida, Spain

**Keywords:** meta-analysis, systematic review, colorectal cancer, survival, epithelial–mesenchymal transition, oxidative stress

## Abstract

**Simple Summary:**

Metastasis is responsible for most of the deaths related to cancer patients. One of the hypotheses that explains the initiation of metastasis is a process called epithelial-to-mesenchymal transition (EMT), in which tumor cells change shape and acquire more aggressive properties that allows them to escape from the tumor and invade other organs. This also occurs in colorectal cancer (CRC), one of the most diagnosed types of cancer worldwide. During the past years, many scientists have discovered that certain molecules or biomarkers participating in this EMT process are able to predict the severity of the cancer and this is helping clinicians to manage treatments. Nevertheless, we think that all this information needs a detailed revision because a lot of biomarkers have been described but have not been analyzed whether they interact with each other in the same mechanism or not. Herein, we performed a bibliographic revision on this topic and identified a great number of biomarkers participating in oxidative stress, a cellular phenomenon that could have a role on the patient’s prognosis because its presence or absence on the patient’s tumor or blood had an influence on survival. Our findings suggest that oxidative stress deserves further study to understand metastasis better and to predict prognosis in a more efficient way.

**Abstract:**

Epithelial-to-mesenchymal transition (EMT) is one of the most accepted mechanisms leading to metastasis, which is responsible for most of the cancer-related deaths. In order to identify EMT-related biomarkers able to predict clinical outcomes in colorectal cancer (CRC), a systematic review and meta-analysis of prognostic factors associated to overall survival (OS) and progression free survival (PFS) was conducted. The systematic literature search included studies from June 2014 to June 2019 available at PubMed and Scopus databases. Meta-analysis was performed for those markers appearing in minimum three works with a total number of 8656 participants. The rest were enlisted and subjected to functional enrichment. We identified nine clinical biomarkers and 73 EMT-related molecular biomarkers associated to OS and/or PFS in CRC. The significant enrichment of biomarkers found involved in cellular oxidoreductase activity suggests that ROS generation plays an active role in the EMT process. Clinical practice needs new biomarkers with a reliable prognostic value able to predict clinical outcomes in CRC. Our integrative work supports the role of oxidative stress in tumorigenesis and EMT progress highlighting the importance of deciphering this specific mechanism to get a better understanding of metastasis.

## 1. Introduction

Colorectal cancer (CRC) is the second most commonly diagnosed cancer type worldwide and is the fourth leading cause of cancer deaths [1]. The prognosis of patients with CRC has greatly improved due to advances in early detection and treatment. However, 30% of patients who undergo curative resection die within a few years after surgery due to metastasis, mainly in the liver [2]. The metastatic process begins with the transition of tumor cells from epithelial characteristics to mesenchymal features (epithelial–mesenchymal transition or EMT). In particular, the EMT program induces disruption of cell adhesions, loss of apical-basal polarity, drastic remodeling of the cytoskeleton and acquisition of mesenchymal cells-related abilities such as the increase in the migratory capacity, leading to invasiveness. This is accompanied by a high resistance to apoptosis and an increase in the production of extracellular matrix components [3,4]. Biomarkers that allow clinicians to distinguish these tumors with high metastatic capacity would help for the therapeutic decision-making process.

The tumor–node–metastasis (TNM) pathological classification is the recommended prognostic tool for CRC [5]. Other factors with strong impact in prognosis are: poorly differentiated or high histological grade, vascular or lymphatic or perineural invasion, intestinal obstruction or perforation at diagnosis and elevated preoperative serum carcinoembryonic antigen (CEA) [6]. In fact, some of these factors serve as indicators of risk assessment in early CRC, which has been categorized into two differentiated groups: low-risk and high-risk groups. The low-risk group is composed by stage I and a fraction of stage II patients who generally undergo curative surgery without adjuvant treatment. The high-risk group encompasses a portion of stage II and the total of stage III patients, who are often treated with adjuvant chemotherapy after tumor resection. Nevertheless, there is an intense debate about the real benefit of using this categorization to guide chemotherapy administration, especially in high-risk stage II patients [7,8]. Moreover, these prognostic factors fail to precisely predict patient’s outcomes due to the wide range of OS rates observed across different stages [9]. Similarly, an individual risk of recurrence after treatment cannot be accurately predicted due to a high variability among individuals [10].

It is known that tumor genetic aberrations such as allelic loss of chromosome 18 [11], microsatellite instability-deficient mismatch repair (MSI/dMMR) [12] and mutations in *KRAS* [13] and in *TP53* [14] have a detrimental effect on prognosis. Regarding EMT biomarkers, low expression of E-cadherin [15] and high expression of N-cadherin [16], Slug and Vimentin [17] have been linked to poorer prognosis in CRC. Multiple molecular signatures have proven to be useful in the stratification of patients according to risk of recurrence [18,19]. However, a lack of large-scale validation and low feasibility of its integration in the clinical practice are common concerns.

There is a high amount of information being generated about prognostic factors in CRC during the past years. However, they offer partial information, about just one or a few biomarkers, without integrating it with previous findings. Thus, the elaboration of an integrative scheme that could amalgamate published data with a quantified effect on overall survival (OS) and progression free survival (PFS) would allow scientists to see the whole scenario.

Herein, we present a comprehensive five-year retrospective systematic review and meta-analysis about prognostic biomarkers related to the EMT pathway. This study focuses on those processes such as oxidative stress signaling and non-coding biology that unravels novel promising prognostic biomarkers.

## 2. Results

### 2.1. Search Results

**Figure 1:**
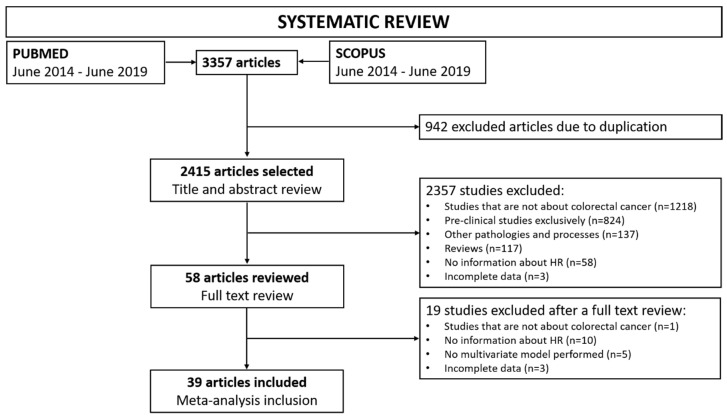
Study flow diagram. Systematic revision workflow from search to included studies for meta-analysis. Exclusion criteria of discarded studies are specified.

A total of 3357 unique indexed citations published between June 2014 and June 2019 were identified. Of these, 58 were selected for evaluation according to the defined eligibility criteria assessed in titles and abstracts. Finally, 39 studies with a total of 8656 participants were included in the meta-analysis and data was extracted after full text assessment. In total, 1219 studies were excluded because they were not about CRC, 824 presented exclusively preclinical data, 137 were related to other pathologies or processes, 117 were reviews, 68 lacked HR determination, 5 did not perform multivariate models and 6 had incomplete data as they were letters to the editor, communications in congresses, etc. The flowchart of the systematic review is presented in Figure 1.

### 2.2. Review of Eligible Studies

Among the 39 publications included, 3 were multicentric and the rest (36) were unicentric studies. All the studies had at least one discovery cohort that was an internal retrospective collection or a repository dataset (The Cancer Genome Atlas (TCGA): https://www.cancer.gov/about-nci/organization/ccg/research/structural-genomics/tcga; and Gene Expression Omnibus (GEO): https://www.ncbi.nlm.nih.gov/geo/). Six studies included validation cohorts, from either TCGA or internal collections. OS, PFS, cancer specific survival (CSS), recurrence-free survival (RFS), metastasis-free survival (MFS) and disease-free survival (DFS) were determined as predicted endpoints. From the total, 22 studies only assessed OS or CSS and two only determined PFS or RFS. Meanwhile, 15 works assessed both OS and PFS.

Pathological staging followed TNM (American Joint Committee on Cancer (AJCC) [20]/Union for International Cancer Control (UICC) [21]) or Dukes [22] classifications. TNM staging was the chosen system in 37 studies, whereas Dukes classification was used in two articles. Most of the works (27) included patients of stage I to IV disease while in five studies, patients cancers were categorized as I to III stages. One study recruited only stage II patients and six studies included metastatic patients exclusively (stage IV).

The cited prognostic biomarkers were determined by protein content: by immunohistochemistry (IHC), immunocytochemistry (ICC) or flow cytometry. Others were assessed by expression profiling such as real-time quantitative PCR (RT-qPCR), in situ hybridization (ISH) or a microarray. Finally, Sanger sequencing was used for polymorphisms identification. The type of sample analyzed was a primary tumor piece in 35 articles and circulating tumor cells (CTCs) in four works. All the information about included studies is specified in Table 1.

### 2.3. Meta-Analysis of the EMT-Related Prognostic Biomarkers in CRC

A meta-analysis was performed for each of the potential EMT-related prognostic biomarkers associated with OS, PFS or both in CRC patients, found in any of the 39 included studies, and found in at least three independent articles. Overall, our findings showed that nine clinical biomarkers were detected to be relevant for prognosis: Pathological tumor extension (pT), pathological node involvement (pN), pathological metastasis (pM), pathological staging (pStage), lymphovascular invasion (LVI), histological grade (HG), tumor size, tumor location and chemotherapy administration. In addition, we identified four molecular prognostic biomarkers that were CEA, CA19.9, Ki67 and E-cadherin. The results from the meta-analysis are summarized in Table 2.

#### 2.3.1. Clinical Prognostic Biomarkers

**Figure 2:**
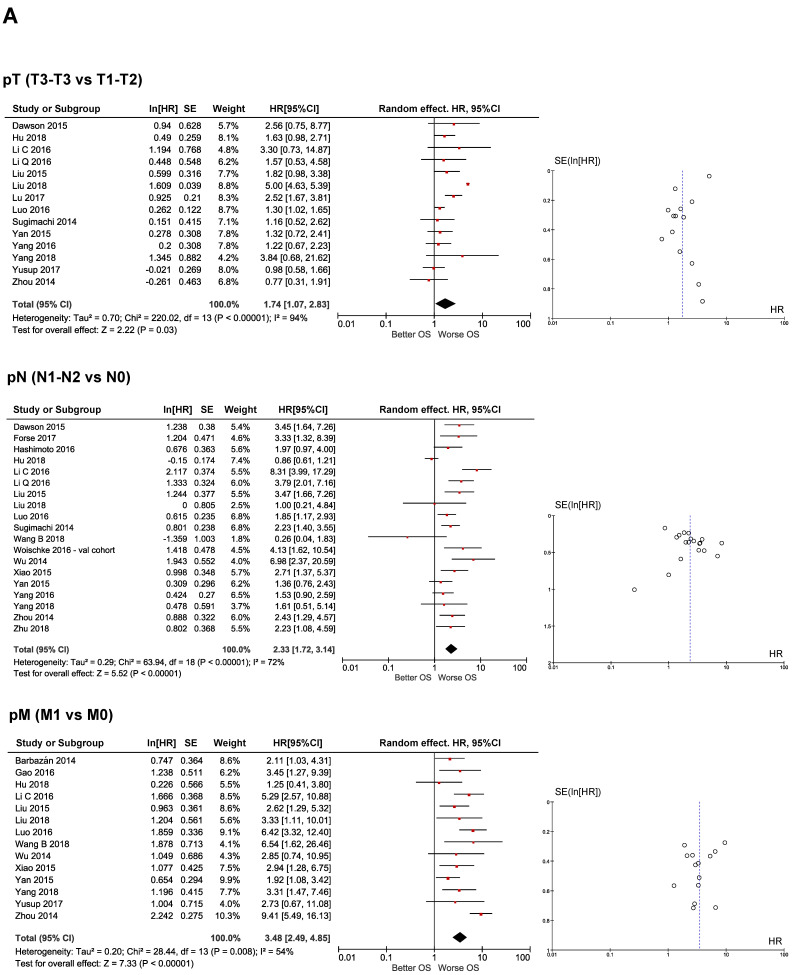

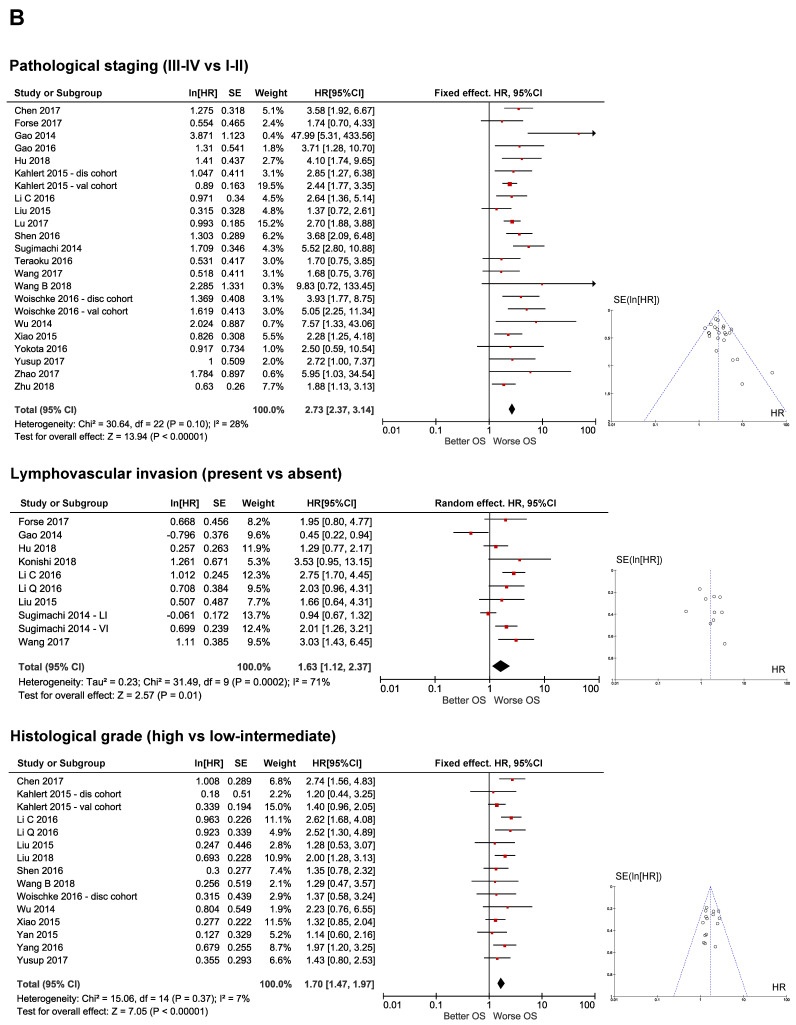

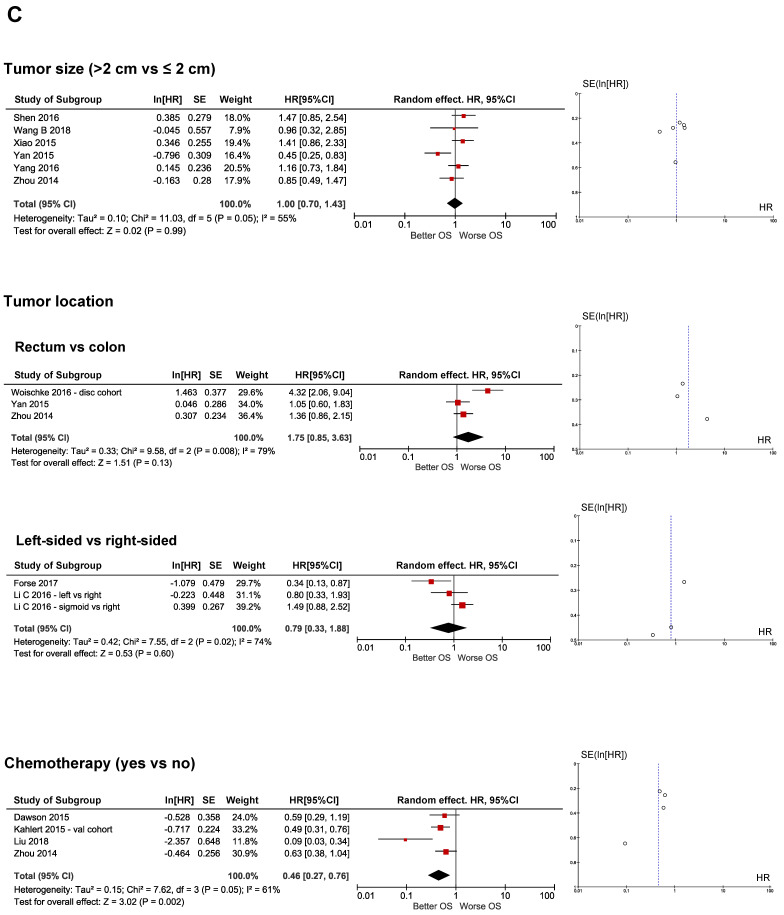

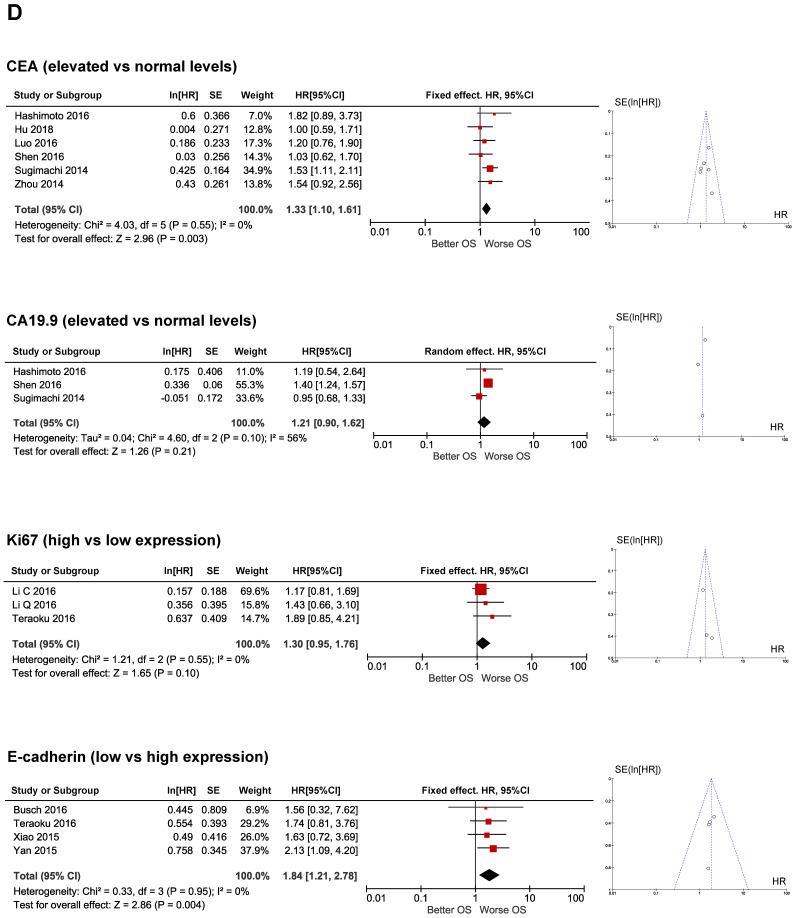
Meta-analysis of EMT-clinical and molecular prognostic biomarkers predicting OS in CRC. (**A**) Forest and funnel plots of pathological T, N and M; (**B**) forest and funnel plots of pathological stage, lymphovascular invasion and histological grade; (**C**) forest and funnel plots of tumor size, tumor location and chemotherapy administration and (**D**) forest and funnel plots of CEA, CA19.9, Ki67 and E-cadherin. Red rombs represent specific HR for each study and their size determines individual weight. Black rombs represent HR global value and their size represents 95%CI. All Studies and Subgroups are referenced in Table 1.

**Figure 3:**
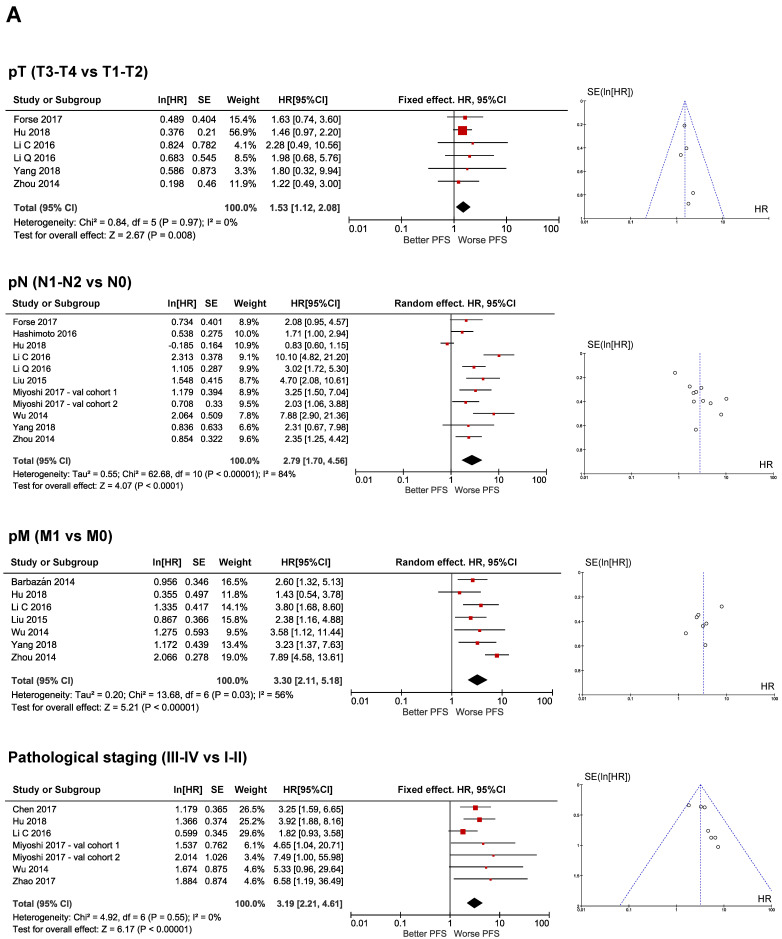

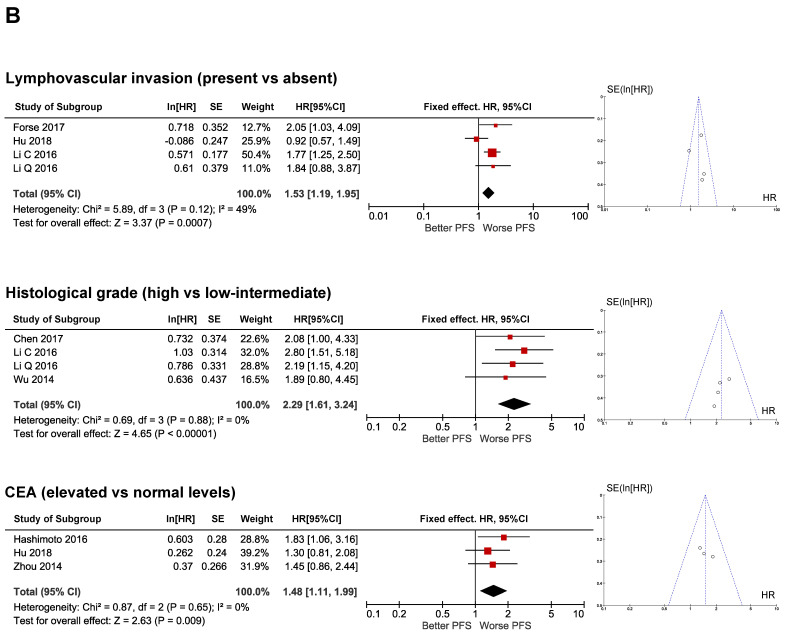
Meta-analysis of EMT-clinical and molecular prognostic biomarkers predicting PFS in CRC. (**A**) Forest and funnel plot of pathological T, N and M and stage and (**B**) forest and funnel plot of lymphovascular invasion, histological grade and CEA. Red rombs represent specific HR for each study and their size determines individual weight. Black rombs represent HR global value and their size represents 95%CI. All Studies and Subgroups are referenced in Table 1.

There were a major number of studies predicting OS than PFS, which was the reason why we found more clinical biomarkers associated to the OS than to PFS. Pathological factors such as pT, pN, pM, pStage, LVI and HG were identified as strongly significantly affecting both OS and PFS (Figure 2A,B and Figure 3A,B), while chemotherapy was only found to be significantly associated to OS. Tumor size and tumor location were not significantly associated to any survival endpoint (Figure 2C). The test of heterogeneity confirmed homogeneity in pStage and HG in predicting both OS (Figure 2A) and PFS (Figure 3A,B), while meta-analysis of pT and LVI were homogenous only in predicting PFS (Figure 3A,B). In these cases, the symmetric distribution of funnel plot shapes demonstrated a low risk of publication bias. In contrast, variables such as pN, pM, tumor location, tumor size and chemotherapy showed heterogeneity in their prediction, suggesting a high risk of publication bias (Figure 2A–C and Figure 3A). Fixed or random effect models were applied accordingly.

#### 2.3.2. Molecular Prognostic Biomarkers

CEA was the unique molecular biomarker identified as an independent prognostic factor of both OS and PFS that was statistically significant (Figure 2D and Figure 3B), while E-cadherin was only significantly associated to OS. CA19.9 and Ki67 were not associated to survival (Figure 2D). In contrast to what happened with clinical biomarkers, meta-analysis results from molecular biomarkers were homogeneous for almost all variables cases except for CA19.9 levels in predicting OS (Figure 2D). Therefore, the most widely used type of effect model was the fixed-effect with results showing a low risk of publication bias as evidenced by the symmetry of their funnel plots.

### 2.4. EMT-Related Molecular Biomarkers in CRC

**Figure 4:**
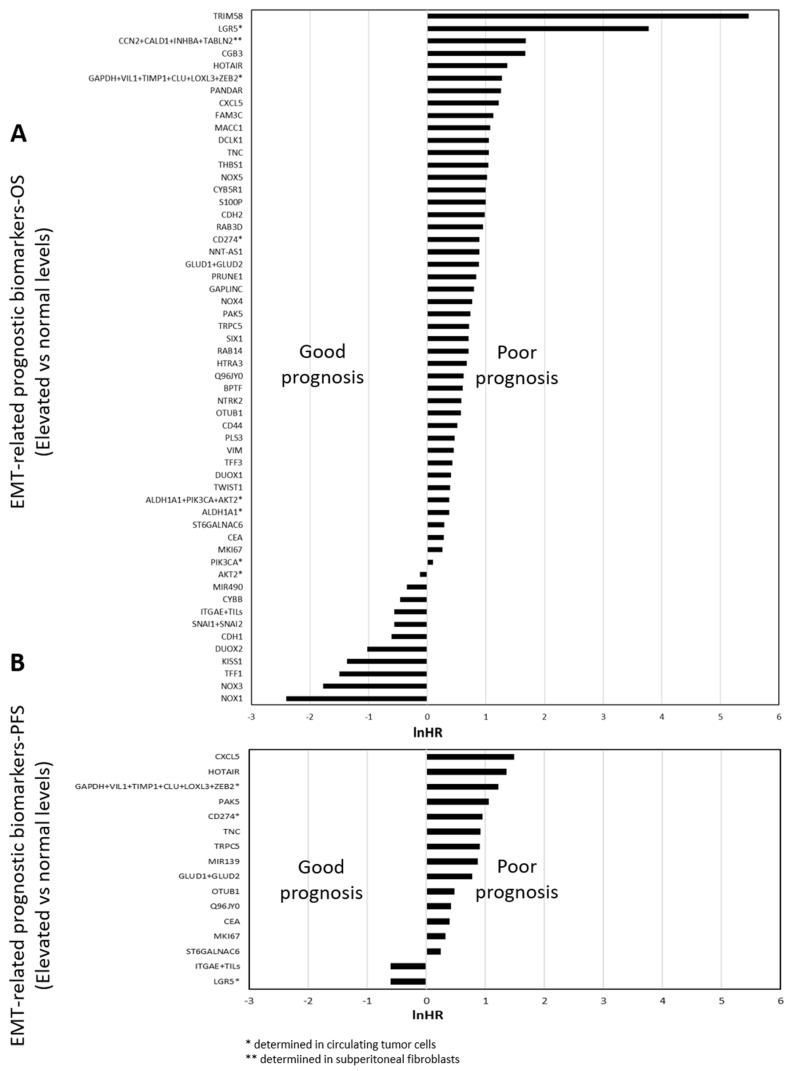
Classification of EMT-related prognostic molecular biomarkers in CRC. (**A**) Molecular biomarkers predicting OS and (**B**) molecular biomarkers predicting PFS.

In total, we found 73 EMT-related molecular biomarkers associated with prognosis in CRC patients, as represented in Figure 4.

Almost all biomarkers found were proteins. Remarkably, we also found the presence of several non-coding RNAs including two microRNA (miRNA): *miR-490-3p* and *miR-139-5p* and four long-non-coding RNA (lncRNA): *HOTAIR, GAPLINC, NNT-AS1* and *PANDAR* (Figure 4).

Of them all, four (CEA, CA19.9, ki67 and E-cadherin) were mentioned in a previous section and analyzed through meta-analysis, and the remaining 69 were only mentioned in one or two independent works. In addition, 24 were independent prognostic factors for OS and PFS, while 48 and 1 were exclusively associated to OS or PFS, respectively (Figure 4).

Regarding the biomarker determination, 69 of them were determined through mRNA or protein levels, while five consisted of specific gene polymorphisms. In order to simplify all the information, Figure 4 shows two bar diagrams containing the 69 biomarkers represented according to its LN (HR), ranging from 5.49 to −2.41 for OS, and from 1.48 and −0.61 for PFS, for the specific comparison of elevated/high levels versus normal/low levels.

### 2.5. Functional Enrichment Analysis

**Figure 5:**
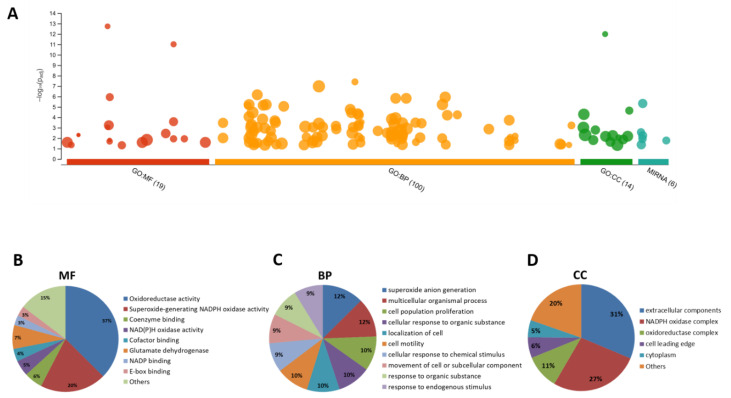
Profiling analysis of EMT-related molecular prognostic biomarkers in CRC. (**A**) Manhattan Plot of the significantly changed terms enriched by GO and MIRNA databases. Circular graphics about the percentages of the specific –log10 (padjust) values of the most represented categories over the total, for: (**B**) molecular function of MF, (**C**) biological process or BP and (**D**) cellular compartment or CC.

Functional enrichment was applied to our biomarker list in order to map genes into known functional information and detect enriched terms. Overall results showed a significant enrichment in several terms regarding GO and MIRNA databases, as seen in the Manhattan plot (Figure 5A). A total of 100, 19 and 14 GO terms were enriched for biological process (BP), molecular function (MF) and cellular compartment (CC), respectively. Additionally, six terms were found in MIRNA databases, containing both miRNA and lncRNA. Detailed classification revealed that oxidoreductase activity, superoxide-generating NADPH oxidase activity and coenzyme binding were the main enriched MF processes (Figure 5B) with a combined percentage of 63%. BP was mainly concentrated in superoxide anion generation, multicellular organismal process and cell population proliferation, with a percentage of 34% (Figure 5C). Additionally, extracellular components, NADPH oxidase and oxidoreductase complexes were the main CC, and their percentages were as high as 64% (Figure 5D). Detailed information about each biomarker categorization is illustrated in Figure S1.

## 3. Discussion

Seven clinical biomarkers, not strictly related to the EMT pathway, were found to be significantly associated to survival: pT, pN, pM, pTNM, LVI, HG and chemotherapy. The local extent of the tumor and the presence of involved nodes are known factors influencing survival. Population-based data of 109,953 N0 patients confirmed that patients with T1–2 cancers had an increased 5-year survival compared to T3, and T3 higher than T4 [61]. This tendency was also maintained in N2. Another study with data from 50,042 patients is also aligned with our findings, showing better survival on those patients with lesions categorized as T1–2 compared to T3–T4, both in N1 and N2 [62]. Additionally, it seems that T stage has a preponderant role on survival where lesions growing into the peritoneum (T4a) have a better prognosis than those invading adjacent tissues (T4b), regardless of the N category [61]. Another work supporting this idea suggests that TNM should be reconsidered by T stage weighting as this affects CRC survival more significantly than the N stage [63]. The presence of metastasis at diagnosis is observed in around 25% of newly diagnosed CRC cases, and 20% of CRC patients will develop distant metastasis during disease course [64]. Distant metastasis is strongly associated to poor outcome in CRC, being the primary cause of treatment failure and consequent death [65]. HG reflects the morphology and proliferative capacity of the primary tumor and has been repeatedly described as an independent prognostic factor in CRC though multivariate model analysis [66]. HG has classically been divided in three distinct categories: well (G1), moderate (G3) and poor (G3) differentiated tumors. G3 tumors are more aggressive and more prone to acquire EMT-like features. Some authors highlight the assessment of the dedifferentiation phenotype and desmoplastic environment, which provides a more individualized outcome prediction than conventional grading and staging systems [67].

The administration of chemotherapy is significantly associated with higher survival. In particular, adjuvant chemotherapy provides significantly higher disease free survival benefit by reducing the rate of recurrence rate and by translating into long-term OS in resected II and III-staged patients [68,69]. Additionally, neoadjuvant chemotherapy provides a benefit in those patients with initially unresectable liver metastasis, impacting both OS and PFS [70].

Regarding LVI, we found that this factor was a significant predictor of both OS and PFS. LVI is considered to be an strong stage-independent prognostic factor and influences decisions regarding the administration of adjuvant chemotherapy in CRC patients with stage II tumors [71]. The presence of tumor cells within the endothelium-lined lymphatic of vascular channels is a very common feature in CRC, accounting for 10-89.5% of the cases [72], which precedes lymph node metastasis and systemic dissemination of cancer cells [73]. In contrast, other authors suggest that vascular invasion rather than lymphatic invasion is responsible for distant recurrences [74].

We did not find significant associations between tumor size and tumor location with survival. There is a great controversy in the literature on these variables. Tumor size, defined as the widest horizontal diameter of tumors, is not involved in the AJCC TNM staging system for CRC as it is in other cancers, and some studies reported no effects on survival [6,75]. Conversely, a more recent study including data from 300,386 patients, concludes that tumor size predicts long-term survival in colon cancer patients subjected to colectomy [76]. Similarly, a study deploying multivariate analysis on 3971 stage I–III CRC patients with curative resection has identified that a tumor size greater than 4 cm is an independent risk factor for CSS [77]. This same study indicates that the tumor side location has a differential impact on OS. Small tumors (≤4 cm) placed on the right side of the colon presented worse prognosis than the ones placed at other locations. There is, in fact, a general agreement in considering that right-sided tumors have worse prognosis than the left-sided counterparts, irrespectively of tumor staging [78,79]. In contrast, other studies suggest that the prognostic value of tumor location depends on other confounding factors such as an elevated systemic inflammatory response and high CD3^+^ immune infiltrate at the tumor margin and within cancer cell nests, especially in resected I–III CRC patients [80].

Regarding molecular prognostic biomarkers, we found that elevated levels or expression of CEA, CA19.9, Ki67 and loss of E-cadherin expression were associated to poor OS, and CEA levels inversely correlate with PFS. CEA is the most used tumor marker in CRC and is involved in cell adhesion and cancer progression shown to target adherens junctions in CRC cell lines [81]. Interestingly, preoperative serum CEA levels correlates with the CEA-cell associate molecule 1 expression in tumors, which induces EMT and tumor angiogenesis in hepatocellular carcinoma [82]. At the clinical level, preoperative serum CEA levels over 5 ng/mL are significantly associated to decreased disease free survival in CRC patients [83] and postoperative levels positively predict recurrence and survival [84]. The tumor antigen CA19.9 is a tetrasaccharide carbohydrate synthetized by the gastrointestinal epithelium and is considered an established serum biomarker for monitoring treatment efficacy in pancreatic cancer patients [85]. Although, the determination of CA19.9 together with CEA adds value to the prognosis, it is still insufficient to manage CRC patients [86]. Preclinical data from CRC cell lines shows a relationship between CA19.9 antigen exposure and metastatic potential through an EMT-related process. In particular, the responsible enzymes for CA19.9 synthesis, fucosyl-transferases, enhance TGF-β signaling resulting in CRC cell migration and invasion, potentiating cancer cell adhesion to endothelial cells by upregulation of Sialyl Lewis antigens [87]. In many cancers such as breast and gastric cancer, nuclear Ki67 positively correlates with tumor grading and is a reliable indicator of tumor recurrence risk [88]. In CRC, however, there is a discrepancy in considering Ki67 as a prognostic marker. While some authors claim that Ki67 labeling index is an independent prognostic factor indicating poor prognosis [89,90], others defend that Ki67 expression is associated with a favorable outcome [91]. Ki67 expression often positively correlates with EMT-related factors such as survivin, vimentin and N-cadherin, thus promoting tumor aggressiveness [92,93]. Finally, E-cadherin is a calcium-dependent glycoprotein localized in the adherens junctions, playing a role in cell adhesion and in maintaining the epithelial morphology [15]. In cancer, loss of E-cadherin caused by mutations, proteolytic cleavage or gene promoter silencing is responsible for invasiveness, anoikis resistance and metastasis dissemination through the EMT program [94]. In CRC, low expression of E-cadherin [16] and the existence of some specific polymorphisms in E-cadherin gene [38] are considered as independent prognostic factors of increased survival.

The impact of non-coding RNAs in CRC prognosis is being deeply explored. Remarkably, the HR values of miRNA and lncRNA were the highest among our entire biomarker list. miRNA are involved in almost all aspects of cancer biology and are considered tumor suppressors or oncogenes depending on the cellular context in which they are expressed [95]. They are former members of the regulatory networks of EMT program like TGF-β/ZEB axis and involved in Notch, Wnt and p53 signaling pathways [96]. They are generally found overexpressed in CRC tissue [97]. Although *miR-139-5p* has been found upregulated in blood and tissue of recurrent CRC patients [49], another work suggests an inhibitory role of this miRNA on EMT in CRC cell lines [98]. We found that downregulation of *miR 490-3p* in CRC tissue correlated with poor prognosis, which is supported by the tumor suppression role of *miR 490-3p* in repressing migration and invasion through partial TGF-β signaling described by others [99].

Regarding the lncRNAs described in our study, they strongly predicted OS when upregulated. Several studies support the role of lncRNAs in the regulation of tumor progression and metastasis through the regulation of the EMT, acting as EMT promoters or EMT suppressors [100]. Specifically, the four lncRNAs identified in our systematic review, *PANDAR, HOTAIR, NNT-AS1* and *GAPLINC*, are independent factors of poor OS when elevated, and *HOTAIR* also predicts poor PFS. Additionally, *HOTAIR* has been shown to promote EMT through the activation of Wnt/Notch signaling and its upregulation constitutes a prognostic factor in esophageal squamous cell carcinoma [101].

It is worth mentioning that the EMT pathway is represented in The Consensus Molecular Subtypes of Colorectal Cancer described in 2015 by Guinney et al. [102]. In his work, authors demonstrated the presence of four different molecular subtypes (CMS1–4) in CRC. In particular, CMS4 tumors had upregulation of genes implicated in EMT and signatures associated with the activation of TGF-β signaling, angiogenesis, matrix remodeling pathways and complement inflammatory system. This subtype is also enriched for downregulated miRNAs (e.g., *hsa-mir-148a*, the *miR-192* and *miR-200* families), and such downregulation is associated with suppression of EMT, matrix remodeling and TGF-β-associated signatures. This could explain the higher aggressivity of CMS4 tumors compared to the other CMS subtypes. At the clinical level, the CMS classification possesses a significant prognostic value in metastatic CRC and according to Mooi et al [103] this seems to be independent from the first-line treatment. In contrast, the FIRE-3 trial demonstrated that CMS classification is predictive for outcomes in CMS4, favoring FOLFIRI plus cetuximab-treated tumors compared to FOLFIRI plus bevacizumab-treated cancers when they are RAS wild type [104].

Our work encourages the analysis of epithelial-like markers such as E-cadherin, which loss of expression has an impact on both OS and PFS in patients treated with oxaliplatin or irinotecan-based chemotherapy, as described in [42]. In addition, the evaluation of mesenchymal-like markers is also worth of attention. In this regard, we suggest the determination of vimentin and N-cadherin expression as they impact on OS, as previously reported [16,31]. However, not adjuvant treatment was specified in these works. Furthermore, the evaluation of EMT promoters such as *TWIST*, *ZEB* and *SNAIL* family members sounds sensible. Related to these markers, it seems that the detection of specific polymorphisms rather than their expression predicts survival. In particular, *TWIST1* polymorphisms predict survival in patients with metastatic CRC receiving first-line bevacizumab plus oxaliplatin-based chemotherapy [38]. To summarize, we thought that the identification of a multiple marker EMT signature based on IHC, qRT-PCR or SNP sequencing methods as routine testing in the anatomy pathology laboratory is highly valuable to predict OS and PFS in CRC patients.

Results from functional enrichment of the molecular biomarker list revealed consistent roles around oxidative stress. Interestingly, oxidoreductase activity accounted for the major GO molecular function annotated and superoxide anion generation for the most demanded BP. Moreover, we would like to emphasize a significant presence of biomarkers in the extracellular compartment or being part of the NADPH oxidase complex. Aerobic respiration generates reactive oxygen species (ROS), which at normally low concentrations are necessary for several cellular processes such as signal transduction, enzyme activation, gene expression, disulfide bond formation and caspase activity control [105]. However, when the antioxidant defense of the cell is overwhelmed and oxidative exacerbates, cell damage takes place, a fact that is considered to be a central event in the physiopathology of several disorders, including cardiovascular and neurodegenerative diseases [106]. In cancer, ROS promotes cellular proliferation, evasion of apoptosis and anoikis, tissue invasion, metastasis and angiogenesis [105]. The NAPDH oxidase complex, where the catalytic component isoforms are Nox1–Nox5 and Duox1–2, is one of the main sources of intracellular ROS when activated. NOX family members regulate redox signaling that ultimately leads to angiogenesis, as reviewed in [107]. In particular, Nox1 upregulates VEGF expression and thus activates VEGF receptors (VEGFR1 and VEGFR2), and hydrogen peroxide production enhances matrix metalloproteinase activity [108]. In addition, it has been shown that Nox4 is a critical regulator of the ROS-mediated DNA damage response induced by oncogenic H-Ras^Val12^, one of the most frequent mutated oncogenes in CRC [109]. ROS is also directly linked to the EMT process through the activation of NF-κB, HIF-1α, TGF-β and extracellular matrix remodeling proteins such as integrins and MMPs, all highly sensitive to the redox status [110]. Overall, a detailed knowledge of redox factors on tumors will add value in the prediction of prognosis with more accuracy.

The main limitation of this meta-analysis is the high diversity of patient’s clinical status across all included studies, which ranges from I to IV stages, and the differences in the treatment management of patients. Although this fact adds variability in our meta-analysis, the extraction of data only from those articles containing HR values adjusted for the individual characteristics of the corresponding study ensures consistency.

## 4. Materials and Methods

### 4.1. Eligibility Criteria for Study Inclusion

We included all the observational studies published from June 2014 to June 2019 involving CRC patients. In order to be included, studies had to evaluate prognostic biomarkers related to the EMT process and to predict patient’s OS and/or PFS. We included biomarker information extracted by any technique (protein, gene expression and sequencing) and from any matrix: Frozen tissue, paraffin tissue and blood. All works contained the adjusted hazard ratio (HR) with its 95% confidence interval (CI) for each biomarker in association with either OS or PFS or both or equivalent, evaluated through multivariate Cox logistic regression. We only included articles published in English. We excluded reviews and original articles containing only preclinical data and studies researching about biomarkers associated to treatment, diagnosis or toxicity. This systematic review had no previously registered protocol.

### 4.2. Literature Search and Systematic Review Procedure

The search strategy was performed in PUBMED and SCOPUS. The search included the following terms:

**1.** “Colonic Neoplasms” [Mesh] OR “Sigmoid Neoplasms” [Mesh] AND “Biomarkers” [Mesh] OR “Biomarkers, Tumor” [Mesh] AND “Epithelial-Mesenchymal Transition” [Mesh] OR “Snail Family Transcription Factors” [Mesh];

**2.** “Colonic Neoplasms” OR “Sigmoid Neoplasms” AND “Biomarkers” OR “Biomarkers, Tumor” AND “EMT” OR “EMT pathway”. Duplicates were removed.

The systematic search of literature was assessed by three investigators (RG, AN and MAS). Disagreements were solved by consensus. Finally, data extraction and synthesis were performed by three investigators (EP, RG and MAS) and included: article identifier, author, year, work design, study cohorts, number of patients in each cohort grouped by the clinical stage, included variables in the multivariate logistic regression model with its adjusted HR with CI for each one of the prognostic biomarkers cited by each study, technique of biomarker determination, predicted variable (OS and/or PFS and/or equivalent) and treatment (neo-adjuvant and/or adjuvant treatment).

This systematic review and meta-analysis followed the published Preferred Reporting Items for Systematic Reviews and Meta-Analyses (PRISMA) [111].

### 4.3. Statistical Analysis of the Meta-Analysis

Hazard ratios (HRs) with the corresponding 95% CI obtained through multivariate logistic regression models were extracted from publications. If needed, the reciprocal HR and CI values were calculated in order to be annealed with the magnitude comparison for each variable. Calculations of Log (HR) and standard error (SE) were performed. The meta-analysis was based on the inverse variance method between the results of at least three independent studies for each variable.

Heterogeneity of included studies was calculated using Higgin’s *I*^2^ index [112] and chi^2^ tests. Heterogeneous studies were considered when *I*^2^ was more than 50% and chi^2^ *p*-value < 0.05. In case of discrepancy between both tests, chi^2^ *p*-value was prioritized. The random effect model was applied on heterogeneous studies. Otherwise, the fixed effect model was used when the studies were homogeneous.

Meta-analysis results were illustrated in forest plots, reporting both weighted and pooled effect from individual studies with their corresponding global HR, 95% CI, and *p*-value.

Possible publication bias was evaluated through visual inspection of funnel plots (i.e., an asymmetrical distribution). The statistical analysis of meta-analysis was performed using Review Manager Software (RevMan-v5.3; Cochrane, Oxford, UK).

### 4.4. GO Profiler Analysis

In those biomarkers in which meta-analysis could not be performed due to a lack of HR estimations available, functional enrichment was executed in order to interpret the resulting biomarker list. The statistical enrichment analysis was carried out by g:Profiler (version e99_eg46_p14_f929183; link: https://biit.cs.ut.ee/gprofiler/gost) and the analysis parameters were as follows: a specific organism was chosen (*Homo sapiens* (human)) and MIRNA and GO analyses (GO molecular function (GO:MF), GO cellular component (GO:CC) and GO biological process (GO:BP)) were carried out sequentially. The statistical domain scope was used only for annotated genes. The significance threshold in the g:Profiler analysis was the g:SCS multiple testing correction method applying significance threshold of 0.05 [113].

## 5. Conclusions

This work identified 9 clinical and 73 EMT-related molecular biomarkers associated to CRC prognosis described in the last 5 years. Apart from the classical ones, novel molecular markers implicated in the EMT process were being considered factors with promising prognostic value. Emerging biomarkers involved in oxidoreductase activity suggest a critical role of ROS in tumorigenesis, particularly in angiogenesis, which is one of most targeted processes in CRC. Although antiangiogenics have become indispensable for the treatment of CRC, more research is needed to identify and validate predictive biomarkers of efficacy. Unraveling ROS mechanisms could provide this valuable information. Furthermore, epigenetic regulation through non-coding RNA in EMT represents a complex framework of interactions that warrant further exploration to understand the process as a whole.

## Figures and Tables

**Table 1:** Characteristics of included studies for meta-analysis. All studies have included colorectal cancer (CRC) patients with described epithelial–mesenchymal transition (EMT)-related clinical and molecular biomarkers predicting overall survival (OS) and/or progression free survival (PFS).

PMID	First Author	Year	Predicted Endpoint	Included Variables in Multivariate Logistic Model	Design	Valitation Cohort	Total Number of Patients	Clinical Stage	Sample/Technique	Tissue Analyzed
24752533	Barbazan [23]	2014	OS, PFS	4-week CTC marker model, baseline CTC marker model, ECOG performance status, lung metastasis	Unicentric	No	50	TNM: IV: 50	CTC/RTqPCR	Colon and rectum
24738665	Gao [24]	2014	OS	ILEI expresssion, TNM stage, peritumoral deposits, lymphatic invasion, venous invasion	Unicentric	No	194	TNM: I: 55; II: 59, III: 74, IV: 6	Tumor/IHC	Colon and rectum
24217791	Sugimachi [25]	2014	OS	Plastin 3 expression, pT, pN, lymphatic invasion, venous invasion, Dukes stage, CEA and CA19.9 levels	Unicentric	No	177	Dukes: A–B: 101; C–D: 76	Tumor and blood/RTqPCR	Colon and rectum
24840737	Wu [26]	2014	OS, MFS	*HOTAIR* gene expression, pN, pM, AJCC stage, HG	Unicentric	No	120	TNM: I: 13, II: 44, III: 49, IV: 14	Tumor/RTqPCR	Colon
25431208	Zhou [27]	2014	OS, PFS	OTUB1 expression, age, gender, tumor location, tumor size, pT, pN, pM, CEA levels, therapy	Unicentric	No	260	TNM: I: 61, II: 63, III: 76, IV: 60	Tumor/IHC	Colon and rectum
25382057	Dawson [28]	2015	OS	mTrkB expression, pT, pN, adjuvant therapy	Unicentric	No	211	TN: I–III: 182, IV: 22, UNK: 7	Tumor/IHC	Colon and rectum
25951369	Kahlert [29]	2015	OS	SIX1 expression, *KRAS* and MSI status, age, gender, HG, UICC stage, therapy	Multicentric	Yes Internal cohort: 817 patients	945	TNM: Cohort 1: I: 42,II: 45, III: 41.Cohort 2: I: 188, II: 314, III: 315	Tumor/IHC, RTqPCR	Colon and rectum
25947346	Liu [30]	2015	OS, PFS	GDH expression, HG, pT, pN, pM, venous invasion, nervous invasion	Unicentric	No	104	TNM: I/II: 42, III/IV: 62	Tumor/IHC	Colon and rectum
25936636	Yan [16]	2015	OS	N-cadherin and E-cadherin expression, age, gender, tumor location, HG, tumor size, pT, pN, pM	Unicentric	No	102	TNM: I–II–III: 53, IV: 49	Tumor/IHC, RTqPCR	Colon and rectum
25716692	Xiao [31]	2015	OS	BTPF expression, vimentin expression, E-cadherin expression, age, gender, tumor size, HG, UICC stage, pN, pM, recurrence	Unicentric	No	105	TNM: I–II: 61, III–V: 44	Tumor/IHC, RTqPCR	Colon and rectum
26507436	Busch [32]	2016	OS	E-cadherin and Snail expression	Multicentric	No	190	TNM: Local: 100 Regional: 66 Distant: 24	Tumor/IHC	Colon and rectum
27520310	Gao [33]	2016	OS	DCLK1 expression, TNM stage, pM	Unicentric	No	71	TNM: I: 11, II: 17, III: 32, IV: 11	Tumor/IHC RTqPCR	Colon and rectum
27037526	Hashimoto [34]	2016	OS, DFS	h-Prune expression, pN, pM, hepatectomy type, CEA and CA19-9 levels	Unicentric	No	87	TNM: IV: 87	Tumor/IHC	Colon and rectum
27537253	Li [35]	2016	OS, PFS	Ki67 and MAEL expression, pT, pN, HG	Unicentric	No	185	TNM: I–II: 105, III: 80	Tumor/IHC	Colon and rectum
27323857	Li [36]	2016	OS, MFS	PAK7 and Ki67 expression, age, gender, location, pT, pN, pM, AJCC stage, vascular invasion, HG	Unicentric	No	203	TNM: I: 24, II: 81, III: 80, IV: 18	Tumor/IHC, RTqPCR	Colon
27046094	Luo [37]	2016	OS	Rab3D expression, pT, pN, pM and CEA levels	Unicentric	No	300	TNM: I: 48, II: 92, III: 103, IV: 7	Tumor/IHC	Colon and rectum
26983880	Matsusaka [38]	2016	OS, PFS	*TWIST1*, *SNAIL*, *ZEB1* and *E-cadherin* gene polymorphisms	Unicentric	No	220	TNM: Beva cohort: IV: 143 Cetu cohort: IV: 77	Tumor/Sanger Seq	Colon and rectum
27503579	Ning [39]	2016	OS	*ALDH1*, *PI3KCA* and *AKT2* gene expression in CTC’s	Unicentric	No	78	TNM: IV: 78	CTC/RTqPCR	Colon and rectum
27363678	Satelli [40]	2016	OS, PFS	CTC counts, PD-L1 expression in CTC’s	Unicentric	No	62	TNM: IV: 62	CTC/Flow cytometry, ICC	Colon
26975699	Shen [41]	2016	OS	S100P expression, CEA and CA19-9 levels, tumor size, HG, TNM stage	Unicentrico	No	125	TNM: I/II: 50 III/IV: 75	Tumor/IHC RTqPCR	Colon
27404020	Teraoku [42]	2016	OS	HG, THBS1, Ki67 and E-cadherin expression	Unicentric	No	94	TNM: IV: 94	Tumor/IHC	Colon and rectum
27120783	Woischke [43]	2016	CSS	CYB5R1 expression, age, gender, location, pT, HG	Unicentric	Yes: TCGA cohort: 457 patients	678	TNM: Dis cohort: I: 1, II: 35, III: 177, IV: 8	Tumor/IHC	Colon and rectum
27259250	Yang [44]	2016	OS	*GAPLINC* gene expression, tumor size, pT, pN, HG	Unicentric	No	180	TNM: I: 13; II: 96, III: 66, IV: 3	Tumor/ISH	Colon and rectum
26370611	Yokota [45]	2016	RFS	*CTGF-CALD1-INHBA-TAGLN* gene expression, *PDLIM5-MAGL1-SPTBN-TAGLN* gene expression and Dukes stage	Unicentric	Yes: Internal cohort: 113 patients	339	Dukes: Dis cohort: A: 41, B: 94, C: 91 TNM: Val cohort: I: 18, II: 45, III: 42, IV: 8	Tumor and SPF/RTqPCR	Colon
28864720	Chen [46]	2017	OS, MFS	TrpC5 expression, AJCC stage, HG	Unicentric	No	127	TNM: I: 17, II: 52, III: 45, IV: 13	Tumor/IHC	Colon
28716573	Forse [47]	2017	OS DFS	sHtrA3 expression, gender, pT, pN, tumor location, lymphovascular invasion	Unicentric	No	172	TNM: II: 172	Tumor/IHC	Colon and rectum
27629879	Lu [48]	2017	OS	*PANDAR* gene expression, pT, TNM stage	Unicentric	No	124	TNM: I/II: 46 III/IV: 78	Tumor/RTqPCR	Colon and rectum
28262692	Miyoshi [49]	2017	RFS	*miR-139-5p* gene expression, pT, pN	Multicentric	Yes: Internal cohorts 1 and 2 = 111 and 139 patients	497	TNM: Dis cohorts: MC: III: 100; TCGA: II: 42, III: 105; Val cohorts: Cohort 1: II: 60, III: 50 Cohort 2: I: 12, II: 67, III: 60	Tumor and blood/RTqPCR	Colon and rectum
27966450	Wang [50]	2017	OS	*NNT-AS1* gene expression, TNM stage, vascular invasion	Unicentric	No	70	TNM: I/II: 36 III/IV: 34	Tumor/RTqPCR	Colon and rectum
28104986	Yusup [51]	2017	OS	TFF1, TFF3, TWIST1 expression, age, gender, histopathological type, HG, pT, pN, pM, TNM stage	Unicentric	No	75	TNM: I: 15, II: 27, III:27, IV: 6	Tumor/IHC	Colon and rectum
28356111	Zhao [52]	2017	OS, DFS	CXCL5 expression, Dukes stage	Unicentric	No	78	TNM I: 15, II: 43, III: 24, IV: 6	Tumor/IHC, RTqPCR	Colon and rectum
29781053	Cho [53]	2018	OS	*NOX1, NOX2, NOX3, NOX4, NOX5, DUOX1, DUOX2* gene expression	Unicentric	No	458	TNM: I: 76, II: 178, III: 129, IV: 65, UNK: 10	Tumor/microaray mRNA expression	Colon and rectum
30100393	Hu [54]	2018	OS, PFS	ITGAE and TIL expression, TNM stage, pT, pN, pM, perineural invasion, vascular invasion, CEA levels	Unicentric	Yes: Internal cohort: 276 patients	1154	TNM: Val cohort: I: 21, II: 81, III: 132, IV: 42	Tumor/IHC	Colon and rectum
29892782	Konishi [55]	2018	OS	hCGBb expression, vascular invasion, tumor budding	Unicentric	No	80	TNM: I–II: 53, III: 27	Tumor/IHC	Colon and rectum
29956813	Liu [56]	2018	OS	TRIM58 expression, age, gender, therapy, HG, pT, pN, pM	Unicentric	Yes: Internal cohort: 152 patients	313	TNM: Val cohort: I: 26, II: 63, III: 59, IV:1, UNK: 3	Tumor/RTqPCR	Colon and rectum
29949050	Wang [57]	2018	OS, PFS	Total CTC count, mesenchymal CTC count, *LGR5* gene expression in CTC	Unicentric	No	66	TNM: I: 31, II: 15, III–IV: 20	CTC/ISH	Colon and rectum
29916545	Wang [58]	2018	OS	*miR-490-3p* and *RAB14* gene expression, age, gender, tumor size, HG, TNM stage, pN, pM	Unicentric	No	50	TNM: I–II: 23, III–IV: 27	Tumor/RTqPCR	Colon and rectum
30170017	Yang [59]	2018	OS, PFS	Tenascin-C expression, age, pT, pN, pM	Unicentric	No	100	TNM: I: 8, II: 38, III: 21, IV: 33	Tumor/IHC	Colon and rectum
30021598	Zhu [60]	2018	OS	MACC1, CD44, TWIST1 and KISS-1 expression, pN, TNM stage	Unicentric	No	212	TNM: I: 69, II: 67; III: 76	Tumor/IHC	Colon

Abbreviations: AJCC: American Joint Committee on Cancer; CSS: cancer specific survival; CTC: circulating tumor cells; DFS: disease free survival; Dis cohort: Discovery cohort; GEO: Gene Expression Omnibus; HG: histological grade; IHC: immunohistochemistry; ICC: immunocytochemistry, MFS: metastasis free survival; OS: overall survival; PFS: progression free survival; RFS: recurrence free survival; SPF: subperitoneal fibroblasts; TCGA: The Cancer Genome Atlas; TNM: Tumor extension-Node-Metastasis; UICC: Union for International Cancer Control; UNK: unknown; Val cohort: Validation cohort.

**Table 2:** Meta-analysis overall results. Table includes statistical results from meta-analysis, heterogeneity test, number of studies/cohorts, number of participants and effect-model applied.

Clinical and Histological Markers	Predicted Variable	HR (95%CI)	Z (*p*-Value)	I^2^ (%)	Heterogeneity *p*-Value	Number of Studies/Cohorts	Number of Participants	Statistical Effect Model
pT (T3–T4 vs. T1–T2)	OS	1.74 (1.07–2.83)	0.03	94	<0.01	14/14	2449	Random
	PFS	1.53 (1.12–2.08)	<0.01	0	0.97	6/6	1196	Fixed
pN (N1–N2 vs. N0)	OS	2.33 (1.72–3.14)	<0.01	72	<0.01	19/19	3453	Random
	PFS	2.79 (1.70–4.56)	<0.01	84	<0.01	10/11	1757	Random
pM (M1 vs. M0)	OS	3.48 (2.49–4.85)	<0.01	54	<0.01	14/14	1968	Random
	PFS	3.30 (2.11–5.18)	<0.01	56	0.03	7/7	113	Random
pStage (III–IV vs. I–II)	OS	2.73 (2.37–3.14)	<0.01	28	0.10	21/23	3769	Fixed
	PFS	3.19 (2.21–4.61)	<0.01	0	0.55	6/7	1054	Fixed
Lymphovascular Invasion (present vs. absent)	OS	1.63 (1.12–2.37)	0.01	71	<0.01	9/10	1638	Random
	PFS	1.53 (1.19–1.95)	<0.01	49	0.12	4/4	836	Fixed
Histological Grade (high vs. intermediate-low)	OS	1.70 (1.47–1.97)	<0.01	7	0.37	14/15	2694	Fixed
	PFS	2.29 (1.61–3.24)	<0.01	0	0.88	4/4	635	Fixed
Tumor location (left vs. right)	OS	0.79 (0.33–1.88)	0.6	74	0.02	2/3	578	Random
Tumor location (rectum vs. colon)	OS	1.75 (0.85–1.88)	0.13	79	0.008	3/3	583	Random
Tumor size (>2 vs. ≤2 cm)	OS	1.00 (0.70–1.43)	0.99	55	0.05	6/6	822	Random
Chemotherapy (yes vs. no)	OS	0.46 (0.27–0.76)	<0.01	61	0.05	4/4	1440	Random
CEA (elevated vs. normal)	OS	1.33 (1.10–1.61)	<0.01	0	0.55	6/6	1225	Fixed
	PFS	1.48 (1.11–1.99)	<0.01	0	0.65	3/3	623	Fixed
CA19.9 (elevated vs. normal)	OS	1.21 (0.90–1.62)	0.21	56	0.10	3/3	389	Random
E–cadherin (low vs. high)	OS	1.84 (1.21–2.78)	<0.01	0	0.95	4/4	491	Fixed
Ki67 (high vs. low)	OS	1.30 (0.95–1.76)	0.10	0	0.55	3/3	482	Fixed

Abbreviations: CI: confidence interval; HR: hazard ratio; OS: overall survival; PFS: progression free survival.

## Supplementary Materials

The following are available online at https://www.mdpi.com/2072-6694/12/11/3330/s1, Figure S1: Detailed biomarker categorization.
