# Supplementary material for: Prognostic Factors Involved in the Epithelial–Mesenchymal Transition Process in Colorectal Cancer Have a Preponderant Role in Oxidative Stress: A Systematic Review and Meta-Analysis
Source: Cancers (Basel). 2020 Nov 11;12(11):3330. doi: 10.3390/cancers12113330 (PMC7697515; doi:10.3390/cancers12113330)
Supplement: Supplementary file 1 [file cancers-12-03330-s001.pdf]

cancers

Supplementary Materials: Prognostic Factors Involved in the Epithelial–Mesenchymal Transition Process in Colorectal Cancer Have a Preponderant Role in Oxidative Stress: A Systematic Review and Meta-Analysis

Eva Parisi, Anabel Sorolla, Robert Montal, Rita González-Resina, Anna Novell, Antonieta Salud and Maria Alba Sorolla

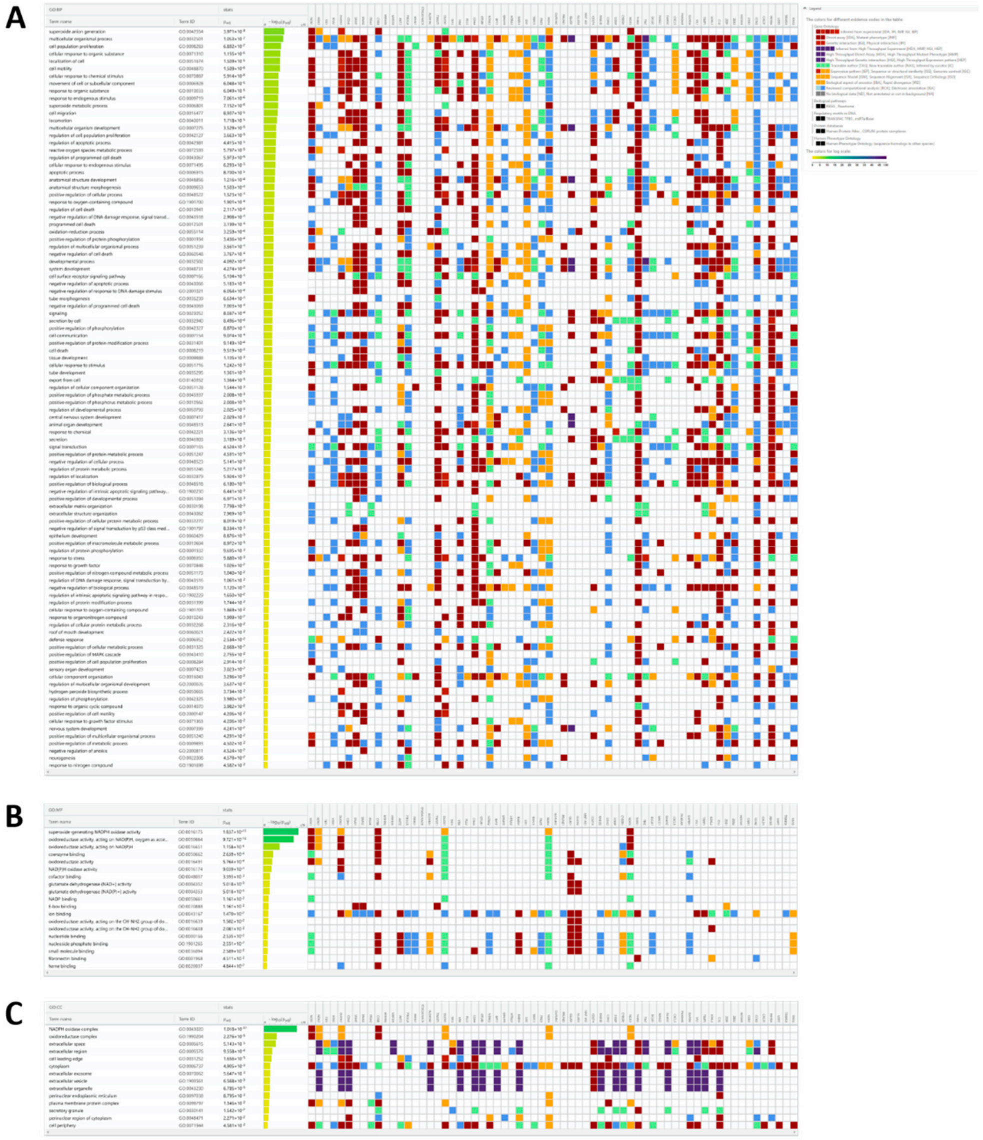

Figure 1. Detailed biomarker categorization.

Publisher's Note: MDPI stays neutral with regard to jurisdictional claims in published maps and institutional affiliations.
